# A multi-center, randomized, clinical study to compare the effect and safety of autologous cultured osteoblast(Ossron™) injection to treat fractures

**DOI:** 10.1186/1471-2474-10-20

**Published:** 2009-02-12

**Authors:** Seok-Jung Kim, Yong-Woon Shin, Kyu-Hyun Yang, Sang-Bum Kim, Moon-Jib Yoo, Suk-Ku Han, Soo-Ah Im, Yoo-Dong Won, Yerl-Bo Sung, Taek-Soo Jeon, Cheong-Ho Chang, Jae-Deog Jang, Sae-Bom Lee, Hyun-Cho Kim, Soo-Young Lee

**Affiliations:** 1 Department of Orthopedic Surgery, Catholic University College of Medicine, Seoul, Korea; 2 Department of Orthopedic Surgery, Dankook University College of Medicine, Cheonan, Korea; 3 Department of Orthopedic Surgery, Konyang University College of Medicine, Daejeon, Korea; 4 Department of Orthopedic Surgery, Inje University College of Medicine, Seoul, Korea; 5 Department of Orthopedic Surgery, Yonsei University College of Medicine, Seoul, Korea; 6 Central Research Institute, Sewon Cellontech, Seoul, Korea

## Abstract

**Background:**

We performed a multicenter, open, randomized, clinical study of autologous cultured osteoblast injection for long-bone fracture, to evaluate the fracture healing acceleration effect and the safety of autologous cultured osteoblasts.

**Methods:**

Sixty-four patients with long-bone fractures were randomly divided into two groups, i.e. those who received autologous cultured osteoblast injection and those who received no treatment. The sum of the difference in the callus formation scores after four and eight weeks, was used as the first efficacy variable.

**Results:**

The autologous cultured osteoblast injection group showed fracture healing acceleration of statistical significance, and there were no specific patient complications when using this treatment.

**Conclusion:**

Autologous cultured osteoblast injection should therefore be considered as a successful treatment option for treating long-bone fracture.

**Trial registration:**

Current Controlled Trials ISRCTN10637905

## Background

During the past few decades, various surgical instruments and external as well as internal metal fixators have been developed for the treatment of fractures; these instruments are constantly being improved in order to provide more effective fracture treatment. To accelerate fracture healing, ultrasound and other treatment methods have also recently been introduced[1]. In particular, cell therapy suggests a new treatment approach[2]. For articular cartilage defects, autologous chondrocyte implantation has become the major treatment[3], and even as a treatment for fracture, the use of autologous cultured osteoblasts has been suggested[4].

When using bone grafts, problems may develop in the donor area in general autologous bone grafts and immunological problems, while the spread of disease may also develop in allografts. There may be faster patient recovery and an absence of these problems when autologous cultured osteoblasts are used[5]. However, as there are only a small number of cells within bone marrow which can be differentiated into osteoblasts[6,7], cell culture is essential for clinical applications. Therefore, it can be anticipated that osteoblasts obtained using cell culture methods may be helpful for healing fractures[8].

Kim et al. showed in animal studies that transplanted autologous cultured osteoblasts induced osteogenesis in bone defect areas[4]. Our current study was conducted to determine whether autologous cultured osteoblasts injected at fracture sites could accelerate the fracture-healing process.

## Methods

### Study patients

This was an open clinical trial, and irregardless of patient gender, among the long-bone shaft (femur, tibia, radius, ulna, humerus) fracture patients between the ages of 15 and 65 years, our study subjects included 64 patients with poor callus formation noted approximately six weeks after surgery (lower than three points on the callus formation score). Fractures were of the closed type and consisted primarily of simple fractures such as transverse fracture or oblique fracture.

Patients deemed unsuitable for study participation and who were therefore excluded included those who were hypersensitive to bovine protein, hypersensitive to gentamycin, those with acute infection in the transplant area, patients positive for HIV, HTLV, HCV, HBV or CMV and on the Syphilis test, pregnant patients, patients who were nursing or who could be pregnant, and patients diagnosed by the investigators to have psychological disorders.

This clinical trial was performed after obtaining permission from the KFDA (Korean Food and Drug Administration). Institutional review board approval and informed consent were obtained from each study patient.

### Bone marrow collection and autologous osteoblast culture

From all patients participating in the clinical trial, during fracture surgery approximately 3 – 5 ml of bone marrow were collected from the anterosuperior iliac spine and were added to a container filled with 30 ml of 10% FBS-α MEM (Sigma Chemical Company, St. Louis, MO, USA) and 350 units of heparin; the mixture was then taken to the GMP institution (Sewon Cellontech, Seoul, Korea).

**Figure 1:**
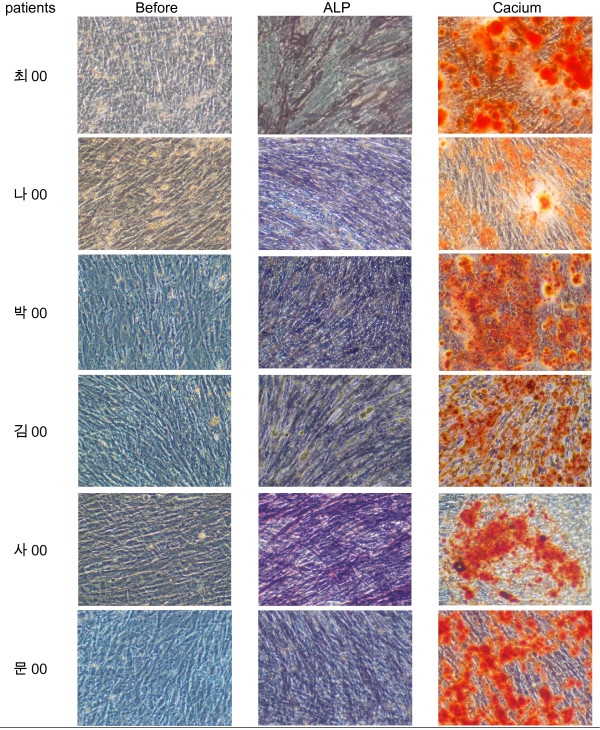
**Before staining, alkaline phosphatase staining, and alizarin red staining at 100 × magnification.(data not shown fully)**.

The mixture was centrifuged at 4°C, 472 g for 10 minutes, after which the supernatant was discarded and 20 ml of culture medium was added to the remaining pellets. The mixture was then filtered (Falcon, Franklin Lakes, NJ, USA), 10 ml of the medium were added per T-75 culture flask (Corning Science Products, Corning, NY, USA), and culture was initiated. The incubator (Automatic CO2 Incubator, Forma Scientific Inc, Marietta, OH, USA) was maintained at 37°C with 5% CO2. The next day, 50 μg L-ascorbic acid (Sigma)/10 ml and dexamethasone 10^-7^M were added to facilitate cell differentiation into osteoblasts. The cell culture condition was evaluated using a light microscope, and the culture medium was changed on the fifth day of culture, after which the culture medium was changed every three days with the subsequent addition of L-ascorbic acid. On the fourteenth day of culture, NBT-BCIP (nitro blue tetrazolium chloride – 5-bromo-4-chloro-3-indolyl phosphate) staining was performed to confirm activation of the alkaline phosphatase(Fig. 1). Twenty-four days after beginning the culture, Alizarin red staining was performed to detect newly produced calcium, and it was thus confirmed that most of the cultured cells were osteoblasts(Fig. 1).

For flow cytometric analysis, monolayer cells were harvested using TrypLE express (Gibco BRL, Gettysburg, PA, USA) and cell numbers were counted using a hematocytometer. Cells were distributed to each test tube at 1 × 10^6 ^and were then resuspended in 50 ul FACS buffer (2% FBS in PBS). For cell permeabilization, Intraprep Reagent (Immunotech, Marseille, France) was used, according to the manufacturer's instructions. Unconjugated, bone-specific monoclonal antibody, Type I collagen (Abcam, Cambridge, UK), and Bone alkaline phosphatase(Abcam, Cambridge, UK) were added, and unspecific staining was determined using isotype controls.

**Figure 2:**
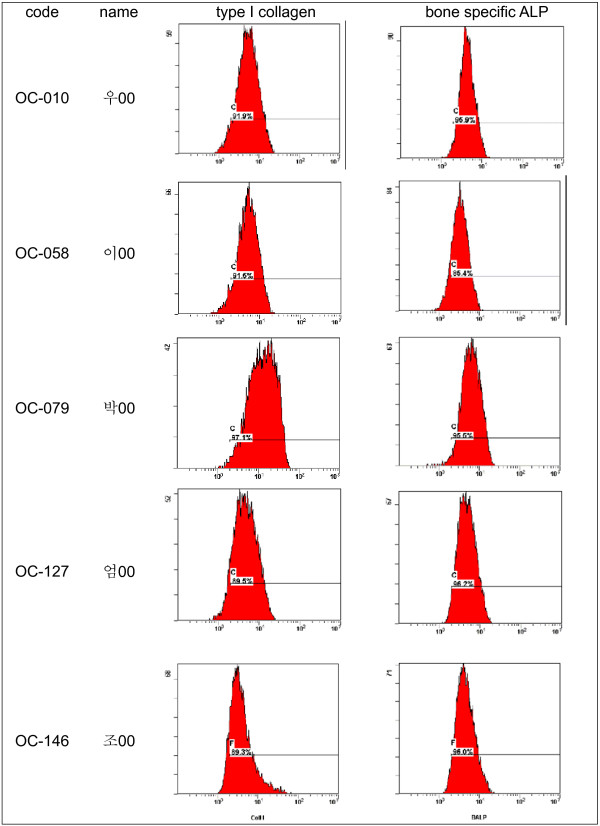
**Type I collagen and alkaline phosphatase expression of cultured cells quantified by FACS analysis.(data not shown fully)**.

To detect fluorescence, FITC conjugated secondary antibody was added to each tube. For analysis, cell pellets were resuspended in FACS buffer and were then stored at 4°C in the dark and were analyzed by flow cytometry within the next 2 h(Fig. 2).

Approximately four weeks after beginning the culture, the medium was removed and the cells were washed with 5 ml 0.02% trypsin-ETDA (Gibco BRL, Gettysburg, PA, USA). 3 ml of 0.02% trypsin-ETDA was added again, and the cells were incubated for five minutes. The trypsin-ETDA activity was stopped by adding 3 ml of culture medium, and all contents were collected in a conical tube and were centrifuged at 4°C, 265 g, for six minutes. The supernatant was removed, and the precipitate was collected. After adjusting the cell count to 1.2 × 10^7^/0.4 ml, the cells(Ossron™, Sewon Cellontech, Seoul, Korea) were used in the transplant.

### Evaluation of patient suitability for study participation (inclusion and exclusion criteria)

This clinical trial was a randomly assigned and open clinical trial designed as a comparative clinical trial and conducted at the Catholic University Hospital, the Young Dong Severance Hospital, the SangGye Paik Hospital, the KonYang University Hospital, and the DanKook University Hospital, all located in Korea. If fracture patients voluntarily agreed to participate in the study, they were enrolled according to both the selection and exclusion criteria. The selection criteria were that approximately six weeks after the first open or closed reduction, the score of the callus formation was lower than three points, and the patient agreed to participate in the clinical trial. Many fractures showed delayed healing when the callus formation score was lower than three points six weeks after the fracture surgery, according to the Catholic University Hospital 2004 fracture patient data. The Korean FDA recommended considering this condition as part of the patient selection criteria in order to prevent unnecessary osteoblast injection.

The exclusion criteria applied to patients whose callus formation score was higher than four points, who had positive results on serum β-HCG testing, who were nursing or possibly pregnant, and who did not consent to participation in the clinical trial. The fracture patterns included primarily simple fracture and, if the radiology examiners were able to evaluate callus formation, also comminuted fractures.

### The experimental and control groups

According to the random assignment table, patients meeting the selection criteria and who were thus enrolled in the study, were divided into the experimental group into whom autologous cultured osteoblasts would be transplanted and the control group. Approximately eight weeks after surgery, autologous cultured osteoblasts were injected into the fracture area of each of the experimental group patients.

### Injection of autologous cultured osteoblasts

Under local anesthesia, each patient was placed on a radiation penetration surgery table; the area to be injected was sterilized according to the surgery preparation procedure. The cultured cells prepared in advance were mixed with fibrin (Greenplast, Green Cross, Korea) at the ratio of 1:1, placed in a syringe, and a 21-G spinal needle was inserted into the syringe. The cell number was 1.2 × 10^7^/0.4 ml in one vial. After five minutes of mixing the cells and fibrin, the viscosity of the mixture had increased. Using a radiation imaging instrument (C-arm), the cells contained in the syringe were injected specifically into the fracture area.

### Follow-up observation after transplant

During the experiment, the patients carefully followed their doctors' instructions and, including their hospitalization, they regularly visited a hospital a total of seven times. To evaluate the safety and efficacy of the osteoblast injection, the experimental group of patients visited a hospital for two months after the transplant of autologous cultured osteoblasts, i.e. at one week, one month, and two months, while the control group visited a hospital at the same times.

### Evaluation methods and statistical analysis

**Figure 3:**
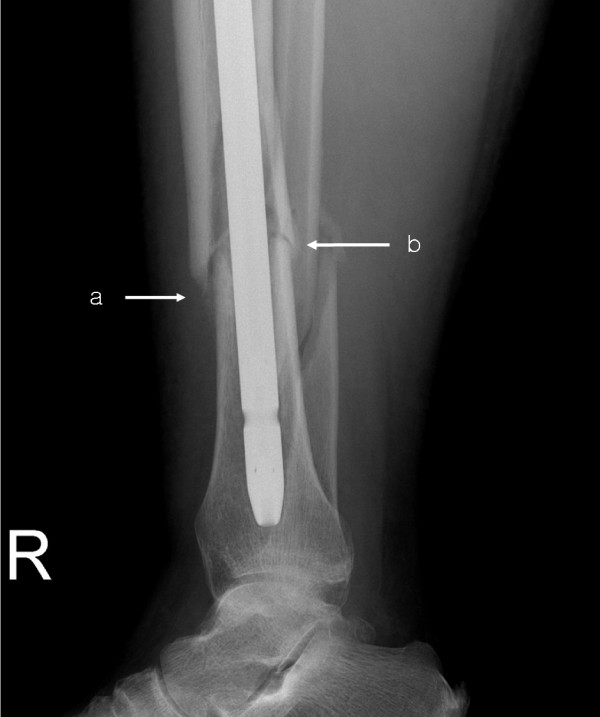
**Method for measuring the callus formation score in the fracture area**. Slight callus formation is given 1 point. b) Bridging callus formation is given 2 points.

To evaluate the effectiveness of the cultured autologous osteoblast injection, the modified callus formation score[9] was used (Fig. 3, Table. 1). No callus formation in one fracture cortex gave 0 points, slight callus formation gave 1 point, and bridging callus formation gave 2 points.

**Table 1:** The method for measuring the callus formation score of the fracture area.

**Point(s)**	**anterior cortex**	**posterior cortex**	**medial cortex**	**lateral cortex**
0	No callus formation	No callus formation	No callus formation	No callus formation
1	Slight callus formation	Slight callus formation	Slight callus formation	Slight callus formation
2	Bridging callus formation	Bridging callus formation	Bridging callus formation	Bridging callus formation

The medial and lateral cortices were evaluated on an anteroposterior radiograph, while the anterior and posterior cortices of the fracture were evaluated on a lateral radiograph. The callus formation scores of each cortex were then totaled. Evaluation of the effectiveness, i.e. evaluation of the callus formation score, was performed by two radiologists, neither of whom had performed the transplant. To obtain the data concerning the effectiveness of the cultured autologous osteoblast injection, PP (Per-protocol) analysis was primarily performed, after which ITT (Intention-to-treat) analysis was used to supplement the PP analysis. The increased callus formation scores one and two months after the cell injection, were thus obtained.

The number of study patients was determined according to the following conditions. The differences in the callus formation scores were averaged between the osteoblast injection time and one month after injection and between the osteoblast injection time and two months after injection. The rationale was that the average callus formation score would differ in both groups. The level of significance was 0.05, type 2 error was 0.2, distribution was 2.13, and the difference value was 1.4. The distribution was based on the data regarding the callus formation score of fracture patients at the Catholic University Hospital. Finally, there were 37 patients in the control group and 35 in the experimental group, anticipating a drop-out rate of 20%. Therefore, the total number of study patients was 72. As many patients were dropped from the study, it was very difficult to accurately determine the exact enrollment number. During the process of enrolling the study patients, this factor became inevitable.

## Results

**Figure 4:**
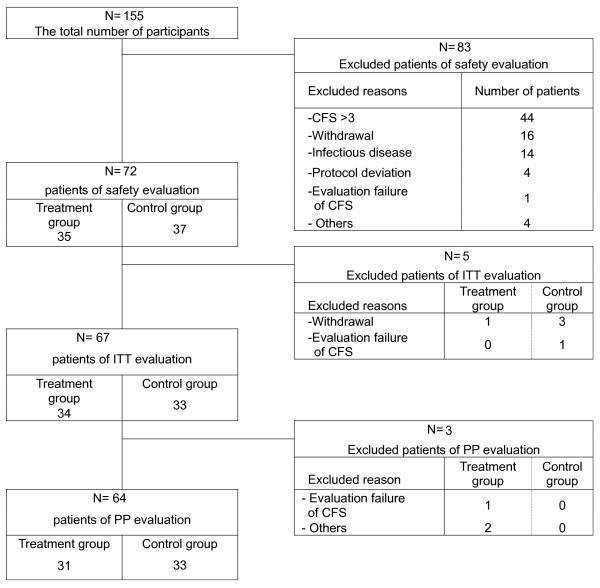
**The recruitment pathways of the clinical trial**.

This clinical trial began on May 13, 2006 and was closed on January 16, 2008. The total number of participants was 155, with 77 enrolled patients less the 13 who dropped out. Therefore, the final total of 64 patients was divided into an experimental group of 31 patients and a control group of 33 patients (Fig. 4).

Of the 78 patients who were excluded, 56.4% were excluded because of a callus formation score greater than 4 points, 12.8% because of their refusal to participate, 20.5% because of positive laboratory findings regarding infectious disease, and 10.3% for various other reasons. There were 21 cases of open reduction and internal fixation (experimental group, 10 cases; control group, 11 cases) and 43 cases of closed reduction and internal fixation (experimental group, 21 cases; control group, 22 cases).

Among the 72 safety evaluation patients, there were 54 males (75.0%) and 18 females (25.0%). In the experimental group, there were 27 males (77.1%) and eight females (22.9%), while there were 27 males (73.0%), and 10 females (27.0%) in the control group. The sex difference between the two groups was not statistically significant (p = 0.68) (Table 2).

**Table 2:** Demographic data.

	Experimental G.	Control G	Sum	p-value
	n (%)	N (%)	n (%)	
Sex				
male	27 (77.1)	27 (72.3)	54 (75)	0.683

female	8 (22.9)	10 (27.0)	18 (25)	χ^2^-test
mean ± std (year)	39.5 ± 12.6	38.6 ± 13.8	39.0 ± 13.2	0.7833
min~max	16 ~65.00	16 ~64.00	16 ~65.00	t-test
Age				
10 ~19	4 (11.4)	5 (13.5)	9 (12.5)	0.8549
20 ~29	4 (11.4)	6 (16.2)	10 (13.9)	Exact test
30 ~39	9 (25.7)	5 (13.5)	14 (19.4)	
40 ~49	12 (34.3)	13 (35.1)	25 (34.7)	
50 ~59	4 (11.4)	6 (16.2)	10 (13.9)	
60 ~69	2 (5.7)	2 (5.4)	4 (5.6)	

Height n(%)	35 (48.6)	37 (51.4)	72 (100)	
mean ± std (cm)	167.7 ± 8.74	168.2 ± 7.55	168.0 ± 8.09	0.817
min~max	151 ~185.00	148 ~190.00	148 ~190.00	t-test

Weight n(%)	35 (48.6)	37 (51.4)	72 (100)	
mean ± std (kg)	66.5 ± 13.7	66.4 ± 10.5	66.4 ± 12.1	0.9837
min~max	39 ~110.00	52 ~100.00	39 ~110.00	t-test

The average patient age in the total patient group was 39.0 ± 13.2 years, while it was 39.5 ± 12.6 years in the experimental group and 38.6 ± 13.8 years in the control group. The age difference between the two patient groups was not statistically significant (p = 0.78). There were 25 patients in their forties (34.7%), 14 (19.4%) in their thirties, and 10 each in their twenties and fifties (13.9%). There was no age distribution difference between the two groups (p = 0.85) (Table 2).

The average patient height was 168.0 ± 8.1 cm, and the average weight was 66.4 ± 12.1 kg; there was no statistically significant difference in either factor between the two groups (p = 0.82, 0.98) (Table 2). There was no statistically significant difference in the fracture distribution between the two groups (p = 0.00) (Table 3).

**Table 3:** The fracture distribution in both groups.

	**Experimental group**	**Control group**
humerus	2	1
radius	1	2
ulna	4	2
femur	11	9
tibia	13	18
fibula	0	1
Sum	31	33

**Figure 5:**
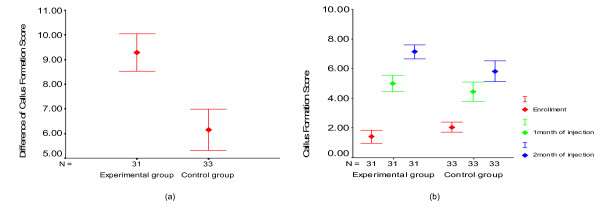
**a) The sum of the difference in callus formation scores in both groups**. b) The callus formation scores in both groups at each time point.

The average sums of the increased amount of callus formation score in the experimental and control groups at one and two months following osteoblast injection, were 9.3 and 6.2, respectively, which was statistically significant (p = 0.00) (Fig. 5a). On the other hand, the average callus formation scores at the time of patient enrollment were 1.4 and 2.1, respectively, in the experimental and control group patients. The final average callus formation scores of the experimental and control groups were 7.1 and 5.8, respectively, which was statistically significant (p = 0.03). But at one month, the data were not statistically significant (p = 0.196) (Fig. 5b). No side effects caused by the osteoblast injection were detected during the clinical trial period.

**Figure 6:**
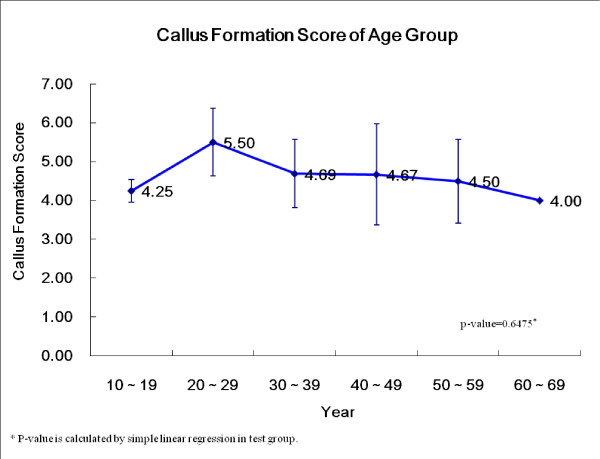
**The callus formation scores according to the patient ages of the osteoblast injection group**.

There was no statistical difference in the osteoblast injection response between the younger and older age groups. Also, when we compared the 20–29 year old group with the other groups, there was no statistical difference (p = 0.71) (Fig. 6).

Adverse events and adverse drug reactions consisted of the usual postoperative findings after fracture treatment, and the adverse reaction rate between the two groups was not statistically different (Table. 4).

**Table 4:** The adverse events.

		Patient number	Occurrence number	p-value
		n (%)	N	
Adverse Event	Exp.	19 (54.3)	42	0.35
	Cont.	16 (43.2)	26	χ^2^-test
	Sum	35 (48.6)	68	

Adverse Drug Reaction	Exp.	9 (25.7)	12	0.19
	Cont.	5 (13.5)	8	χ^2^-test
	Sum	14 (19.4)	20	

Severe Adverse Event	Exp.	1 (2.9)	1	0.49
	Cont.	0 (0.0)	0	Exact test
	Sum	1 (1.4)	1	

There was one MRSA infection in an experimental group patient, and this infection was treated with antibiotics. Our IRB determined that this infection had no relationship to this clinical trial.

## Discussion

Time has increasingly become the most important factor in clinical decision-making. Numerous efforts have been made in various clinical areas to allow trauma patients to return to their normal life as soon as possible and without complications[10]. In particular, in patients with fractures, the fractures generally eventually heal, however, in many patients, bone union can be delayed to an extent that requires bone transplant. Not only does this cause psychological and physical pain to the patient, but on a large scale, it also causes great social loss. The purpose of our study is to attempt to determine how to reduce this loss and to assess the possibility of early recovery using cell therapy without surgery, such as bone transplant, that is painful and may have undesirable aftereffects.

**Figure 7:**
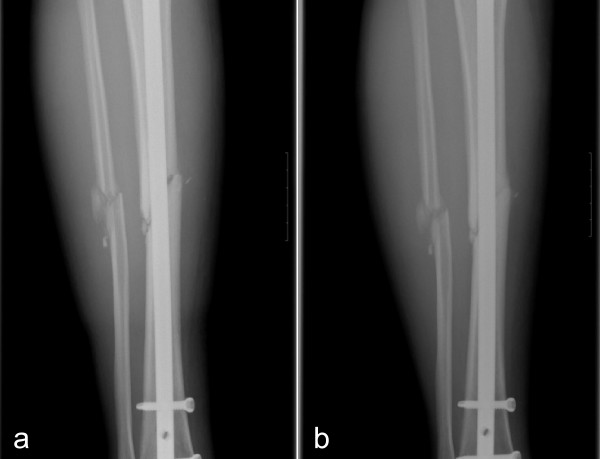
**The right tibia AP radiographs of a 47-year-old male patient before osteoblast injection (a) and eight weeks after injection (b) into the fracture of the tibia shaft**.

With regard to the time required for osteoblast injection, although it varies depending on the patient for cases without osteogenesis, even two months after bone fracture injury when the overall bone union rate was evaluated, the callus formation was relatively slow; in addition, this is the time period during which the osteogenesis activity after fracture decreases[11]. Based on the assumption that irregardless of the type of procedure, if the activity within a fracture could be increased, the effect of the continuous callus formation could be maintained and the optimal time for osteoblast transplant would be approximately two months after the initial treatment. Following transplant, rapid callus formation could be detected in some of our control group patients, however, in general, more rapid and effective callus formation was observed in the experimental patient group(Fig. 7).

Autologous cultured osteoblast injection is based on bone marrow injection, the benefit of which is supported by the theory that osteoprogenitor cells in bone marrow induce and facilitate bone formation[12]. Bone marrow injection is performed independently or is combined with a bone graft procedure. Differing from autologous bone transplant, as bone marrow injection is not a surgical procedure performed by making an incision in the skin in the donor area, its great advantage is that there are no complications or adverse side effects. However, as only a limited amount of bone marrow can be collected from one site, the number of osteoprogenitor cells contained in the aspirated bone marrow is very limited [13]. Therefore, it has been proposed that culturing cells and their subsequent transplantation is the most feasible way to overcome this limitation[6].

There are several reasons why successful osteogenesis achieved by injection of autologous cultured osteoblasts, should be considered, e.g. the procedure is performed by injection using only a syringe and without any incision being made, there is only a small amount of tissue injury, and the procedure does not impair the blood supply.

Because of the surgery required for autologous bone transplant or allogenic bone transplant patients, tissue damage or impaired blood circulation may occur; osteogenesis or bone union may also occur because of the resorption process of transplanted bones. On the other hand, following injection, as autologous cultured osteoblasts are connected to adjacent tissue while undergoing formation of the bone matrix, they do not go through such a process. In our study, autologous cultured osteoblasts were grafted to the bone defect area using fibrin as it not only permits them to safely attach to the defect area but it is also well known that fibrin, which has been used in surgery for the purpose of hemostasis, is safe and is readily absorbed without inducing the reaction caused by foreign material. In addition, fibrin becomes a vehicle to transport growth factors in the area where maintenance of the transplant volume is difficult[14].

The advantages of the technique of autologous cultured osteoblasts are that, in general, as the initial fracture surgery is performed under general anesthesia, additional anesthesia is not required for the bone marrow collection. In addition, as at the time of transplant, cultured osteoblast injection can be performed under local anesthesia of the fracture area. Therefore, following the injection, patients can immediately return to their daily routine and do not require hospitalization.

For the union of bones, autologous bone transplant is certainly the most rapid and effective method, however, considering the pain in the donor area caused by the surgery, the limited volume of the bone graft, and the additional surgery required for transplant, we believe that osteoblast transplant that achieves bone union may be a successful alternative to autologous bone transplant. In addition, if such osteoblast transplants could be used for all fracture patients, not only an early return to their daily routine but also the prevention of complications following surgery, such as delayed union and non-union, could also be anticipated.

## Conclusion

Autologous cultured osteoblast transplant is a safe and effective method for accelerating the rate of fracture healing.

## Abbreviations

CFS: callus formation score; PP: Per-protocol; ITT: Intention-to-treat.

## Competing interests

The authors declare that they have no competing interests.

## Authors' contributions

SK was involved in collecting patient information, reviewing the literature, and drafting the manuscript as the main author. YS, KY, SK, MY, SH, YS, and TJ were involved in the surgery and patient care. SI and YW were involved in the radiologic evaluation. CC is the senior author and was responsible for the final proofreading of the manuscript. JJ, HK, SL, and SL were involved in collecting patient information and in the statistical evaluation. All authors read and approved the final manuscript.

## Pre-publication history

The pre-publication history for this paper can be accessed here:


